# Cardiovascular-Specific Mortality among Gastrointestinal Stromal Tumor Patients: A Population-Based Analysis

**DOI:** 10.1155/2023/3619306

**Published:** 2023-02-14

**Authors:** Hailing Yao, Huiying Shi, Mengke Fan, Lanlai Yuan, Rong Lin

**Affiliations:** ^1^Department of Gastroenterology, Union Hospital, Tongji Medical College, Huazhong University of Science and Technology, Wuhan 430022, China; ^2^Department of Otorhinolaryngology, Union Hospital, Tongji Medical College, Huazhong University of Science and Technology, Wuhan 430022, China

## Abstract

**Background:**

The overall risk of cardiovascular mortality (CVM) in cancer survivors has increased with time. The trend of CVM in patients with gastrointestinal stromal tumors (GISTs) remains unclear. This study is aimed at assessing the risks and independent predictors of CVM in GIST patients.

**Methods:**

Data of the GIST patients were extracted from the Surveillance, Epidemiology, and End Results (SEER) database (2000-2019). The standardized mortality ratio (SMR) was used to evaluate the risk of CVM, and a multivariate competing risk model was utilized to identify the predictors for CVM.

**Results:**

A total of 12,058 patients with GIST were included in this study, of whom 477 (4.0%) patients died of cardiovascular disease (CVD). The SMR for CVM among GIST patients was significantly higher than in the general population (SMR, 3.23, 95% CI: 2.97-3.52), and all categories of CVD were associated with a significantly elevated SMR. The cumulative mortality of CVD was the lowest among all causes of death, while the CVM was the second most common cause of death in patients ≥ 80 years when stratified by age at diagnosis. Furthermore, male sex, older age at diagnosis, White race, unmarried, earlier year of diagnosis, and not receiving chemotherapy were the poor prognostic factors for CVM.

**Conclusions:**

The CVM risk in GIST patients was significantly higher relative to the general US population. Timely screening and cardioprotective interventions should be implemented to prevent the occurrence of CVM in patients with GIST.

## 1. Introduction

Gastrointestinal stromal tumors (GISTs) are the most common malignant subepithelial lesions of the gastrointestinal (GI) tract. Most GISTs originate from Cajal cells, which are the pacemaker cells of the GI tract. GISTs can occur in any part of the GI tract, but most are located in the stomach or small intestine [1, 2]. With advancements in diagnostic technologies, the incidence of GISTs has been increasing steadily over the last few decades, with a global incidence of 10-15 cases per million [3, 4]. The introduction of imatinib and other tyrosine kinase inhibitors as therapies has resulted in considerable increases in overall and progression-free survival in patients with GISTs, with 5-year overall and cause-specific survival rates of 74% and 82%, respectively [5–8].

As life expectancy and cancer survival rates increase, the population of cancer survivors is steadily increasing. As previously reported, the overall risk of cardiovascular disease (CVD) in cancer survivors has increased with time [9–12]. Oh et al. reported a 20-fold increase in cardiovascular mortality (CVM) in cancer patients from 2000 to 2016 in Korea [13]. The risk of CVM in gastroenteropancreatic neuroendocrine neoplasm patients was 1.2 times higher than that in the general US population [14]. Felix et al. demonstrated that the risk of CVM in endometrial cancer patients was 8.8-fold higher compared to women in the general population [15].

Given that there was no study evaluating the risk of CVM in patients with GIST, the aims of our study were to describe cause-specific mortality rates and identify independent predictors of CVM in GIST patients based on the data from the largest database. We expect that results from our study will emphasize the need to intervene early in CVD and thereby further improve survival rates in patients with GIST.

## 2. Methods

### 2.1. Data Source

We extracted patient data from the Surveillance, Epidemiology, and End Results (SEER) database, which covers approximately 34.6% of the US population (http://seer.cancer.gov/). SEER^∗^Stat software (version 8.4.0.1) was used to access the SEER Research Plus Data, 17 Registries (excl AK), Nov 2021 Sub (2000-2019). The cases used to calculate standardized mortality ratios (SMRs) come from the SEER Research Plus Data, 17 Registries (excl AK), Nov 2021 Sub (2000-2019) for SMRs. Data are publicly available and do not require ethical approval for analysis. Authors obtained Limited-Use Data Agreements from SEER.

### 2.2. Study Population

**Figure 1:**
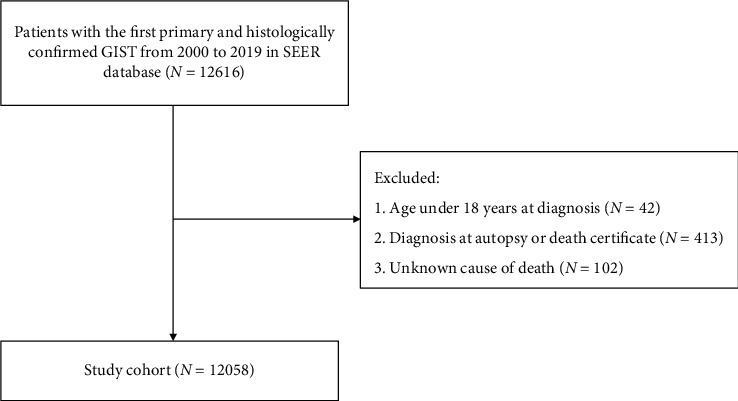
Flowchart of the patient selection criteria.

We collected patients diagnosed with the first primary GIST identified by the primary sites esophagus, stomach, small intestine, colon, rectum, appendix, and peritoneum and histologic code “8935/3.” Patient selection is outlined in Figure 1.

### 2.3. Study Variables and Outcomes

Patient demographics and clinical characteristics, including age at diagnosis, race, sex, primary tumor site, year of diagnosis, marital status, SEER stage, surgery, chemotherapy, radiotherapy, cause of death, survival time, and outcome, were extracted. The survival time was calculated as the time from the diagnosis of cancer to death of the patient due to any cause or to the last follow-up.

The cause of death was categorized by the International Classification of Diseases-10 (ICD-10) codes. The main endpoint was CVM, defined as deaths due to diseases of the heart (I00-I09, I11, I13, I20-I51), hypertension without heart disease (I10, I12), cerebrovascular diseases (I60-I69), aortic aneurysm and dissection (I71), and other diseases of the arteries, arterioles, and capillaries (I72-I78) [16, 17]. Table S1 includes the types of CVM and corresponding codes in the ICD-10.

### 2.4. Statistical Analysis

The risk of CVM for GIST patients was compared to that of the US general residents, and the results were presented as the SMR. SMR was calculated as the ratio of the observed deaths to the expected death via SEER^∗^Stat software. The Poisson exact method was used to compute the 95% confidence interval (CI) and corresponding *p* values for the SMR. Fine and Gray's competing risk regression was performed to account for the two competing events: CVM and non-CVD deaths, and the crude cumulative mortality function was used to express the probability of developing primary and competing events. Multivariate competing risk survival analyses were performed to identify independent predictors of CVM. All analyses were performed using R software (version 4.1.0). A 2-sided *p* value < 0.05 was considered statistically significant.

## 3. Results

### 3.1. Patient Characteristics

A total of 12,058 patients with GIST identified in the SEER database between 2000 and 2019 were included in this study. The distribution of age at diagnosis was 16.3% (18-49 years), 34.4% (50-64 years), 37.3% (65-79 years), and 12.1% (≥80 years). The majority of patients were male (52.0%), White (68.0%) and had local tumor stage (49.5%). The most common tumor location was the stomach (59.1%), followed by the small intestine (27.9%). Of the included patients, 9822 (81.5%) patients underwent surgery, 5088 (42.2%) patients received chemotherapy, and only 116 (1.0%) patients underwent radiotherapy. The mean duration of follow-up was 67.0 months (range, 1-239 months). During the follow-up period, 477 (4.0%) patients died of CVD. Disease of the heart (75.1%) and cerebrovascular diseases (18.2%) were the most common categories of CVM. Patient characteristics are presented in Tables 1 and 2.

### 3.2. Comparison of CVM in Study Participants versus the General Population

The SMR for CVM among GIST patients was 3.23 (95% CI: 2.97-3.52) (Table 1). In the subgroup analysis stratified by different clinical characteristics, the patients with a primary site in the stomach, small intestine, or rectum and no history of radiotherapy had significantly elevated SMRs relative to the general US population, regardless of sex, age at diagnosis, race, marital status, year of diagnosis, stage, surgery, and chemotherapy.

SMRs specific to different categories of CVM are illustrated in Table 2. Overall, patients with GIST had a higher risk of death from CVD than the general population. The highest SMR was seen in the category of other diseases of the arteries, arterioles, and capillaries (SMR, 10.35, 95% CI: 2.82-26.49), followed by aortic aneurysm and dissection (SMR, 3.58, 95% CI: 1.54-7.04) and cerebrovascular diseases (SMR, 3.28, 95% CI: 2.62-4.04).

### 3.3. Cumulative Mortality of CVD

**Figure 2:**
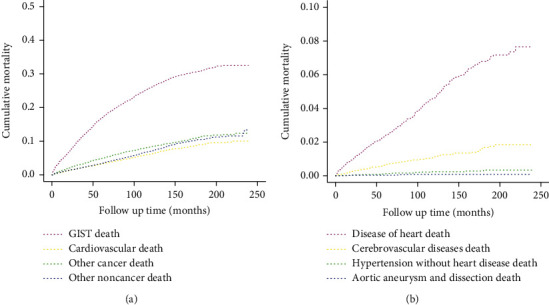
Cumulative mortality for all causes of death (a) and main causes of CVD (b) in GIST patients.

The results from the Fine-Gray model of competitive risk used to assess cumulative mortality for all causes of death in GIST patients are shown in Figure 2(a). The cumulative mortalities for GIST, CVD, other cancer, and other noncancer diseases at 200 months of follow-up were 32.08%, 9.55%, 11.83%, and 11.22%, respectively. The cumulative mortality of diseases of the heart (7.17%) was the highest, followed by cerebrovascular diseases (1.84%) and hypertension without heart disease (0.34%) at 200 months of follow-up (Figure 2(b)).

**Figure 3:**
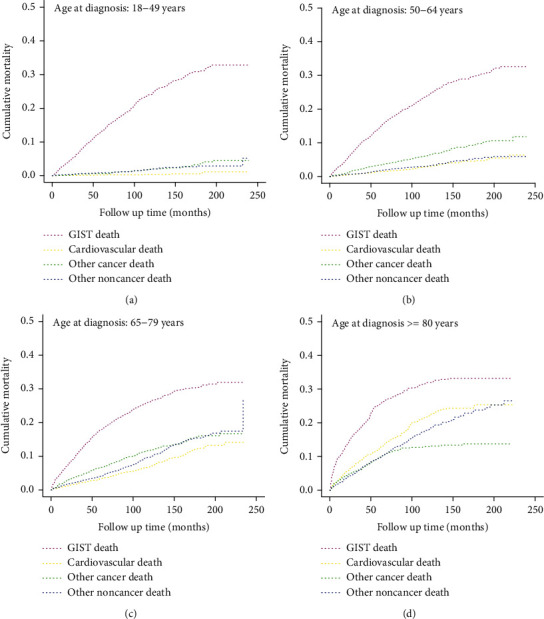
Cumulative mortality for all causes of death in GIST patients stratified by age at diagnosis: (a) age between 18 and 49; (b) age between 50 and 64; (c) age between 65 and 79; (d) age ≥ 80.

**Figure 4:**
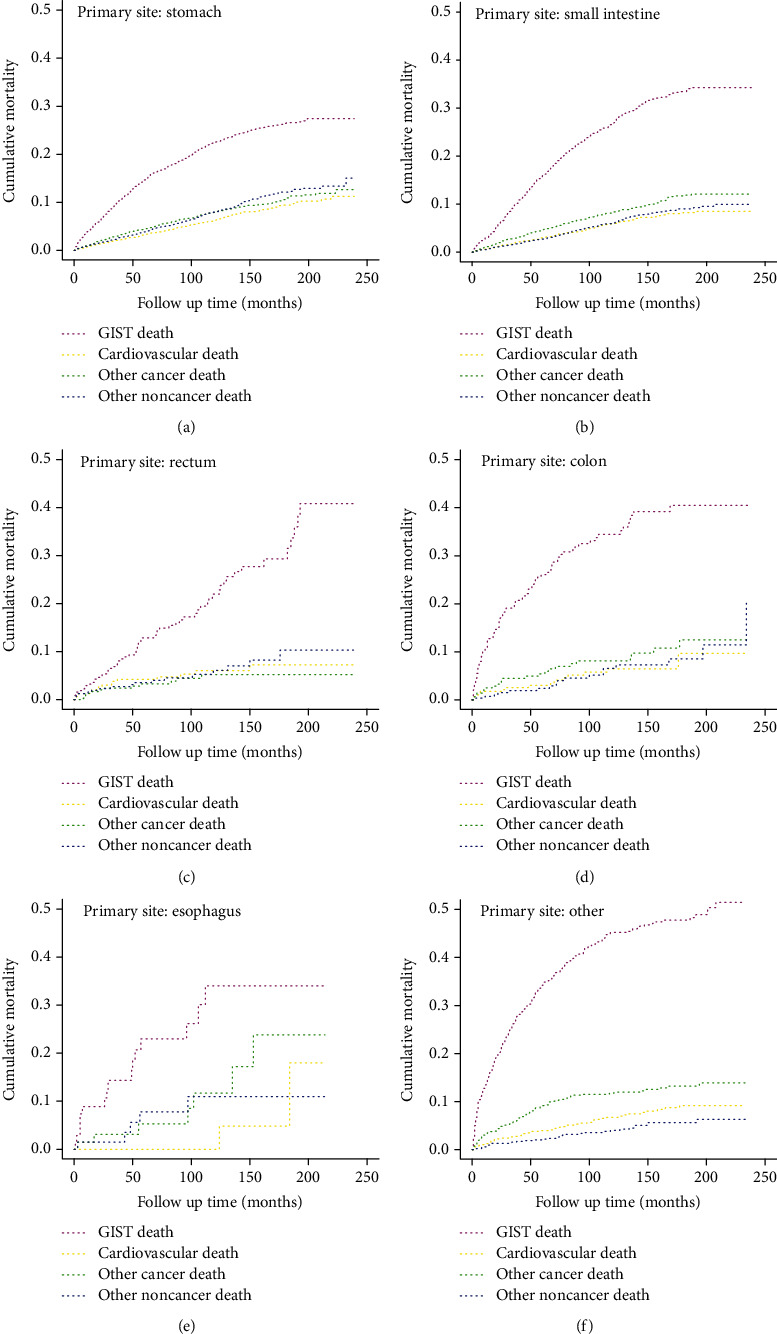
Cumulative mortality for all causes of death in GIST patients stratified by primary site: (a) stomach; (b) small intestine; (c) rectum; (d) colon; (e) esophagus; (f) other.

Figure 3 showed the cumulative mortality stratified by age at diagnosis. The cumulative mortality of CVD increased gradually with age at diagnosis and reached a plateau at approximately 200 months of follow-up. The cumulative mortality of CVD was the lowest among all causes of death when age < 80 years (Figures 3(a)–3(c)), while the cumulative mortality of CVD exceeded that of other cancer and other noncancer deaths in the oldest group (≥80 years) (Figure 3(d)). All patients were stratified by primary site (Figure 4), and the results suggested that esophageal and rectal patients had the highest (17.99%) and lowest (7.22%) cumulative mortality of CVD, respectively (Table S2).

### 3.4. Predictors of CVM

We performed a multivariate competing risk analysis to identify the independent predictors of CVM in GIST patients, and the results are shown in Table 3. Female sex (hazard ratio (HR): 0.803, 95% CI: 0.682-0.947) was independently associated with lower risks of CVM. CVM in GIST patients increased as patients' age increased, with HRs of 6.856 (95% CI: 3.339-14.080), 17.403 (95% CI: 8.605-35.195), and 54.817 (95% CI: 27.075-110.985) for patients aged 50-64, 65-79, and ≥80, respectively, compared to patients aged 18-49. Compared with White patients, there was no significant difference in the risk of CVM for Black patients, whereas the risk of CVM for other ethnicities (American Indians/AK Native and Asian/Pacific Islander) was significantly lower compared with White patients (HR: 0.622, 95% CI: 0.462-0.838). In addition, CVM decreased with the year of diagnosis; patients diagnosed between 2015 and 2019 had a 51.5% decrease (HR: 0.485, 95% CI: 0.360-0.654) in CVM compared to patients diagnosed between 2000 and 2004. Compared with patients with local disease, the risk of CVM in patients with distant disease (HR: 0.684, 95% CI: 0.505-0.925) was significantly reduced. In addition, patients who were not receiving chemotherapy treatment (HR: 1.272, 95% CI: 1.039-1.557) were at a higher risk of CVM than patients receiving chemotherapy treatment.

## 4. Discussion

To the best of our knowledge, this is the first large population-based study to determine the risk of CVD in GIST patients and to identify the predictive factors of CVM in this population. Our results showed that 477 (4.0%) GIST patients diagnosed from 2000 to 2019 died of CVD, and the risk of CVM was 3.23 (95% CI: 2.97-3.52) times that of the general US population. According to the competing risk analysis, we found that the cumulative mortality of CVD was the lowest among all possible causes of death involved. The cumulative mortality of diseases of the heart ranked first among the categories of CVD during the follow-up period. In addition, we identified sex, age at diagnosis, race, marital status, year of diagnosis, and chemotherapy as independent predictors of CVM in GIST patients.

GISTs are largely caused by varying molecular changes in the tyrosine kinase receptor KIT or platelet-derived growth factor receptor-*α* (PDGFRA); the former is considered the “classic GIST” and comprises 75% of GISTs, and the latter is the second most common type and comprises 10% of all cases. Patients with KIT or PDGFRA mutations are sensitive to the tyrosine kinase inhibitor imatinib, which was approved by the FDA in 2002 [4, 18]. Over the past decade, imatinib has been evaluated in several randomized trials and resulted in overall survival benefits in completely resected or metastatic GIST patients [19–21]. Worryingly, imatinib has been reported to have cardiac toxicities [22–24]. Accordingly, we found that patients who underwent chemotherapy had a higher SMR (SMR, 4.77, 95% CI: 4.10-5.54) than those in the general US population. However, information regarding details of the chemotherapy are not available in the SEER database, and therefore, further investigation is required to clarify the contribution of specific chemotherapy to CVM.

In terms of the year of diagnosis, our study showed that the HRs of CVM for patients diagnosed with GIST in 2000-2004, 2005-2009, 2010-2014, and 2015-2019 decreased consistently across time. The key factor underlying this finding might be that advances in imaging techniques and biomarkers in cardiovascular disease enabled earlier detection and intervention [25]. In addition, thalidomide and metformin have been shown to have potential cardioprotective effects, preventing the cardiotoxicity of chemotherapeutic agents at the human and animal levels [26, 27].

In the present study, we found that the cumulative mortality of CVD increased with patient age at diagnosis and accounted for the second most common cause of death in the oldest age group (≥80 years). Furthermore, multivariate competing risk analysis suggested that patients diagnosed at an older age were predisposed to die due to CVD (HR: 54.817, 95% CI: 27.075-110.985). Interestingly, the SMR of CVM was the highest when patients aged 18-49 (SMR, 15.91, 95% CI: 9.19-27.53), similar to the results reported by Sun et al. [14]. We hypothesized that older patients were likely to suffer from worse overall health, decreased immunity, and increased comorbidities such as infection [28], so they might not have a sufficient life expectancy to develop and die of CVD. In addition, we observed that male or unmarried patients had a high probability of CVM compared to controls, which might be due to worse personal lifestyle habits and worse socioeconomic status, as previous studies have proven that smoking, drinking, or living alone play an important role in the development of CVD [29–31].

Our study has some limitations. First, this study was a retrospective analysis of a large database from the US general population, and potential selection bias could not be eliminated, although we used strict screening criteria. Second, the mitotic index, genetic mutations, personal life history of alcohol and tobacco use, and subgrouping the no/unknown group for the radiotherapy and chemotherapy data were not available in the SEER database. In addition, there is a small amount of missing data for certain parameters.

## 5. Conclusions

In summary, our study demonstrates that the SMR of CVM in GIST patients was 3.23 times higher than that of the general US population, and all categories of CVD were associated with a significantly elevated SMR, of whom the cumulative mortality of diseases of the heart ranked first. Competing risk analysis identified sex, age at diagnosis, race, marital status, year of diagnosis, and chemotherapy as independent predictors of CVM in GIST patients. This research has provided evidence for timely screening for CVD in patients with GIST and insight to help clinicians make informed decisions about which GIST patients warrant long-term monitoring for the prevention of CVM.

## Figures and Tables

**Table 1:** Standardized mortality ratios of cardiovascular disease mortality among GIST patients according to baseline characteristics.

Characteristics	Total diagnosis^1^ (%)	CVD^2^		
		Observed deaths (%)	Expected deaths	SMR (95% CI)
Total	12058 (100.0)	477 (100.0)	147.48	3.23 (2.97-3.52)
Sex				
Male	6268 (52.0)	251 (52.6)	71.55	3.51 (3.12-3.94)
Female	5790 (48.0)	226 (47.4)	75.93	2.98 (2.63-3.37)
Age at diagnosis				
18-49	1961 (16.3)	7 (1.5)	0.44	15.91 (9.19-27.53)
50-64	4145 (34.4)	93 (19.5)	8.35	11.14 (9.48-13.09)
65-79	4495 (37.3)	201 (42.1)	58.22	3.45 (3.03-3.93)
≥80	1457 (12.0)	176 (36.7)	80.45	2.19 (1.89-2.53)
Race				
White	8197 (68.0)	346 (72.5)	117.93	2.93 (2.65-3.24)
Black	2136 (17.7)	86 (18.0)	18.71	4.60 (3.79-5.57)
Other	1725 (14.3)	45 (9.4)	10.84	4.15 (3.17-5.43)
Marital status				
Married	9475 (78.6)	388 (81.3)	129.98	2.99 (2.72-3.28)
Unmarried	1947 (16.1)	69 (14.5)	13.76	5.01 (4.06-6.20)
Unknown	636 (5.3)	20 (4.2)	3.74	5.35 (3.62-7.91)
Year of diagnosis				
2000-2004	1965 (16.3)	147 (30.8)	63.69	2.31 (1.97-2.70)
2005-2009	2517 (20.9)	149 (31.2)	50.98	2.92 (2.51-3.41)
2010-2014	3497 (29.0)	132 (27.7)	27.98	4.72 (4.04-5.51)
2015-2019	4079 (33.8)	49 (10.3)	4.83	10.14 (8.09-12.72)
SEER stage				
Local	5965 (49.5)	269 (56.4)	87.48	3.07 (2.75-3.44)
Regional	1337 (11.1)	61 (12.8)	26.28	2.32 (1.82-2.96)
Distant	1670 (13.8)	56 (11.7)	9.81	5.71 (4.53-7.20)
Unknown	3086 (25.6)	91 (19.1)	23.90	3.81 (3.15-4.61)
Primary site				
Stomach	7126 (59.1)	284 (59.5)	95.03	2.99 (2.68-3.34)
Small intestine	3362 (27.9)	123 (25.8)	32.87	3.74 (3.17-4.41)
Rectum	318 (2.6)	12 (2.5)	2.69	4.46 (2.66-7.48)
Colon	292 (2.4)	12 (2.5)	6.85	1.75 (1.00-3.06)
Esophagus	70 (0.6)	2 (0.4)	0.41	4.91 (0.59-17.73)
Other	890 (7.4)	44 (9.2)	9.63	4.57 (3.49-5.98)
Surgery				
Yes	9822 (81.5)	358 (75.1)	122.19	2.93 (2.65-3.23)
No	2139 (17.7)	115 (24.1)	23.94	4.80 (4.07-5.67)
Unknown	97 (0.8)	4 (0.8)	1.35	2.96 (1.17-7.54)
Chemotherapy				
Yes	5088 (42.2)	138 (28.9)	28.96	4.77 (4.10-5.54)
No/unknown	6970 (57.8)	339 (71.1)	118.52	2.86 (2.58-3.17)
Radiotherapy				
Yes	116 (1.0)	4 (0.8)	1.71	2.34 (0.64-5.98)
No/unknown	11942 (99.0)	473 (99.2)	145.77	3.24 (2.98-3.53)

^1^Database “SEER Research Plus Data, 17 Registries (excl AK), Nov 2021 Sub (2000-2019)” was used. ^2^Database “SEER Research Plus Data, 17 Registries (excl AK), Nov 2021 Sub (2000-2019) for SMRs” was used. Race: other (American Indians/AK Native and Asian/Pacific Islanders); primary site: other (appendix and peritoneum). Abbreviation: GIST: gastrointestinal stromal tumor; SMR: standardized mortality ratio; CI: confidence interval.

**Table 2:** The standardized mortality ratios of all causes of cardiovascular mortality in patients with GIST.

CVD	Observed deaths (%)	Expected deaths	SMR (95% CI)
Disease of the heart	358 (75.1)	111.49	3.21 (2.89-3.56)
Hypertension without heart disease	20 (4.2)	6.81	2.94 (1.79-4.53)
Cerebrovascular diseases	87 (18.2)	26.55	3.28 (2.62-4.04)
Aortic aneurysm and dissection	8 (1.7)	2.24	3.58 (1.54-7.04)
Other diseases of the arteries, arterioles, and capillaries	4 (0.8)	0.39	10.35 (2.82-26.49)

Abbreviation: GIST: gastrointestinal stromal tumor; CVD: cardiovascular disease; SMR: standardized mortality ratio; CI: confidence interval.

**Table 3:** Multivariate competing risk analysis for predictors of cardiovascular mortality in patients with GIST.

Characteristics	Adjusted HR	95% CI	*p*
Sex			<0.01
Male		Ref	
Female	0.803	0.682-0.947	
Age at diagnosis			<0.001
18-49		Ref	
50-64	6.856	3.339-14.080	<0.001
65-79	17.403	8.605-35.195	<0.001
≥80	54.817	27.075-110.985	<0.001
Race			<0.01
White		Ref	
Black	1.147	0.917-1.436	0.23
Other	0.622	0.462-0.838	<0.01
Marital status			0.019
Married		Ref	
Unmarried	1.421	1.112-1.815	<0.01
Unknown	1.073	0.720-1.599	0.73
Year of diagnosis			<0.001
2000-2004		Ref	
2005-2009	0.752	0.608-0.931	<0.01
2010-2014	0.587	0.470-0.733	<0.001
2015-2019	0.485	0.360-0.654	<0.001
SEER stage			0.069
Local		Ref	
Regional	0.866	0.669-1.121	0.27
Distant	0.684	0.505-0.925	0.014
Unknown	1.040	0.784-1.378	0.79
Primary site			0.72
Stomach		Ref	
Small intestine	1.120	0.922-1.360	0.26
Rectum	1.281	0.755-2.175	0.34
Colon	0.980	0.602-1.596	0.94
Esophagus	0.549	0.132-2.277	0.41
Other	0.987	0.677-1.438	0.94
Surgery			0.40
Yes		Ref	
No	1.113	0.877-1.412	0.38
Unknown	1.551	0.714-3.371	0.27
Chemotherapy			0.02
Yes		Ref	
No/unknown	1.272	1.039-1.557	
Radiotherapy			0.44
Yes		Ref	
No/unknown	1.469	0.551-3.913	

Race: other (American Indians/AK Native and Asian/Pacific Islanders); primary site: other (appendix and peritoneum). Abbreviation: GIST: gastrointestinal stromal tumor; HR: hazard ratio; CI: confidence interval.

## Data Availability

The datasets used and/or analyzed during the current study are available from the SEER database freely or the corresponding author on reasonable request.

## Abbreviations

CVM: Cardiovascular mortality
GISTs: Gastrointestinal stromal tumors
SEER: Surveillance, Epidemiology, and End Results
SMR: Standardized mortality ratio
CVD: Cardiovascular disease
GI: Gastrointestinal
ICD-10: International Classification of Diseases-10
CI: Confidence interval
HR: Hazard ratio
PDGFRA: Platelet-derived growth factor receptor-*α*.

## Supplementary Materials

Supplementary Materials Supplement Table 1: definition of the included types of cardiovascular mortality and corresponding codes in the ICD-10. Supplement Table 2: cumulative mortality stratified by age at diagnosis and primary site at 200 months of follow-up.
