# Supplementary material for: Cardiovascular-Specific Mortality among Gastrointestinal Stromal Tumor Patients: A Population-Based Analysis
Source: Oxid Med Cell Longev. 2023 Feb 14;2023:3619306. doi: 10.1155/2023/3619306 (PMC9943598; doi:10.1155/2023/3619306)
Supplement: Supplementary Materials — Supplement Table 1: definition of the included types of cardiovascular mortality and corresponding codes in the ICD-10. Supplement Table 2: cumulative mortality stratified by age at diagnosis and primary site at 200 months of follow-up. [file 3619306.f1.docx]

Supplement Table 1. Definition of included types of cardiovascular mortality and corresponding codes in the ICD-10

| Description | ICD-10 | Definition of CVM |
| --- | --- | --- |
| Diseases of heart | I00-I02 | Acute rheumatic fever |
|  | I05-I09 | Chronic rheumatic heart diseases |
|  | I11 | Hypertensive heart disease |
|  | I13 | Hypertensive heart and renal disease |
|  | I20-I25 | Ischemic heart diseases |
|  | I26-I28 | Pulmonary heart disease and diseases of pulmonary circulation |
|  | I30-I32 | Diseases of pericardium |
|  | I34-I39 | Nonrheumatic valve disorders |
|  | I40-I41 | Myocarditis |
|  | I42-I43 | Cardiomyopathy |
|  | I44-I45 | Conduction disorders |
|  | I46 | Cardiac arrest |
|  | I47-I49 | Arrythmias |
|  | I50 | Heart failure |
|  | I51 | Complications and ill-defined descriptions of heart disease |
| Hypertension without heart disease | I10 | Essential (primary) hypertension |
|  | I12 | Hypertensive renal disease |
| Cerebrovascular diseases | I60-I62 | Nontraumatic intracranial haemorrhage |
|  | I63 | Cerebral infarction |
|  | I64 | Stroke, not specified as haemorrhage or infarction |
|  | I65-I66 | Occlusion and stenosis of precerebral/cerebral arteries, not resulting in cerebral infarction |
|  | I67-I69 | Other cerebrovascular diseases or Sequelae of cerebrovascular disease |
| Aortic aneurysm and dissection | I71 | Aortic Aneurysm and Dissection |
| Other diseases of arteries, arterioles, and capillaries | I72 | Other aneurysm |
|  | I73 | Other peripheral vascular diseases |
|  | I74 | Arterial embolism and thrombosis |
|  | I77 | Other disorders of arteries and arterioles |
|  | I78 | Diseases of capillaries |

Abbreviation: ICD-10, International Classification of Diseases-10; CVM, cardiovascular mortality;

Supplement Table 2. Cumulative mortality stratified by age at diagnosis and primary site at 200 months follow-up

| Characteristics | Cumulative morality of all causes of death | | | |
| --- | --- | --- | --- | --- |
|  | GIST | CVD | Other cancer | Other non-cancer diseases |
| Age at diagnosis |  |  |  |  |
| 18-49 | 32.84 | 1.14 | 4.55 | 2.87 |
| 50-64 | 31.84 | 5.48 | 10.64 | 5.93 |
| 65-79 | 31.45 | 13.25 | 16.12 | 16.88 |
| ≥80 | 33.16 | 25.32 | 13.76 | 25.29 |
| Primary site |  |  |  |  |
| Stomach | 27.44 | 10.26 | 11.57 | 12.92 |
| Small intestine | 34.27 | 8.48 | 12.11 | 9.55 |
| Rectum | 40.84 | 7.22 | 5.20 | 10.33 |
| Colon | 40.50 | 9.68 | 12.48 | 11.43 |
| Esophagus | 34.04 | 17.99 | 23.79 | 10.98 |
| Other | 48.90 | 9.18 | 13.90 | 6.27 |

Abbreviation: GIST, gastrointestinal stromal tumor; CVD, cardiovascular disease.
